# 910 metagenome-assembled genomes from the phytobiomes of three urban-farmed leafy Asian greens

**DOI:** 10.1038/s41597-020-00617-9

**Published:** 2020-08-25

**Authors:** Aditya Bandla, Shruti Pavagadhi, Ashwin Sridhar Sudarshan, Miko Chin Hong Poh, Sanjay Swarup

**Affiliations:** 1 Singapore Center for Environmental Life Sciences Engineering, National University of Singapore, Singapore, Singapore; 2 NUS Environmental Research Institute, National University of Singapore, Singapore, Singapore; 3 Department of Biological Sciences, National University of Singapore, Singapore, Singapore; 4 Synthetic Biology for Clinical and Technological Innovation, National University of Singapore, Singapore, Singapore

**Keywords:** Agriculture, Metagenomics

## Abstract

The genome sequences of many microbial species from the phytobiomes of several leafy Asian greens remain unknown. Here, we address this gap by reconstructing 910 prokaryotic draft genomes from 24 leaf, 65 root, 12 soil, and 6 compost metagenomes from the seedling and adult developmental stages of three leafy Asian greens – *Brassica rapa* var. *parachinensis*, *Brassica oleracea* var. *alboglabra* and *Amaranthus* spp. – grown in a commercial, soil-based urban farm. Of these, 128 are near-complete (>90% completeness, <5% redundancy), 540 are substantially complete (≥70% completeness, <10%, redundancy), while the rest have a completeness ≥50% and redundancy <10%. The draft genomes together span 292 bacterial and 3 archaeal species, a subset of which are from underrepresented genus-level lineages in public databases. We expect our dataset to facilitate a wide range of comparative studies that seek to understand the different functional aspects of vegetable crop phytobiomes and for devising new strategies for microbial cultivation in the future.

## Abstract (summary)

**Table:** 

Measurement(s)	Metagenome • metagenomic data • soil metagenome • sequence_assembly • leaf metagenome • root metagenome • compost metagenome
Technology Type(s)	DNA sequencing • sequence assembly process
Factor Type(s)	green leafy vegetable type • growth stage • plant organs
Sample Characteristic - Organism	Brassica rapa • Brassica oleracea • Amaranthus
Sample Characteristic - Environment	urban biome • farm • soil

Machine-accessible metadata file describing the reported data: 10.6084/m9.figshare.12472847

## Background & Summary

Microbiomes within the phytobiome^1^ – the plant, its environment, and its associated communities of organisms – affect nearly all aspects of growth such as development, differentiation, nutrient acquisition, and tolerance to biotic and abiotic stresses^2^. Previous studies have greatly expanded our understanding of the diversity and composition of specific phytobiome-associated microbiomes^3–9^, but only a few have investigated their genetic underpinnings in a systematic manner^10,11^. Knowledge of the latter is especially critical for improving our ability to manipulate phytobiome-associated microbiomes with a view to enhance crop productivity and agricultural sustainability. Metagenomic strategies used to gain such insights rely on curated and well-referenced catalogs of microbial reference genomes that have been specifically recovered from phytobiome-associated microbiomes. Using a catalog of 3,837 bacterial reference genomes, 1,160 of which were from a limited number of phytobiomes, Levy *et al*.^10^ identified genetic traits associated with bacterial adaptation to the phytobiome. Deeper insights into other aspects such as identifying the functional roles of different microbial species within the phytobiomes of specific crops will, however, require access to an expanded catalog of microbial reference genomes recovered from crop phytobiomes of interest.

Leafy Asian greens which include a range of Brassicas and Amaranthus are widely consumed in Asia and are rich in phytochemicals with known health benefits^12^. They are well suited for cultivation in urban farms^13^, where microbiome-based solutions can be readily test-bedded in comparison to trials in large, conventional agricultural farms. Although leafy Brassicas represent the nearest commercial crops to the model plant Arabidopsis, their microbiomes remain poorly understood in comparison to the latter. Similarly, the microbiomes of low-cost leafy vegetables such as Amaranthus, also remain poorly understood.

**Fig. 1:**
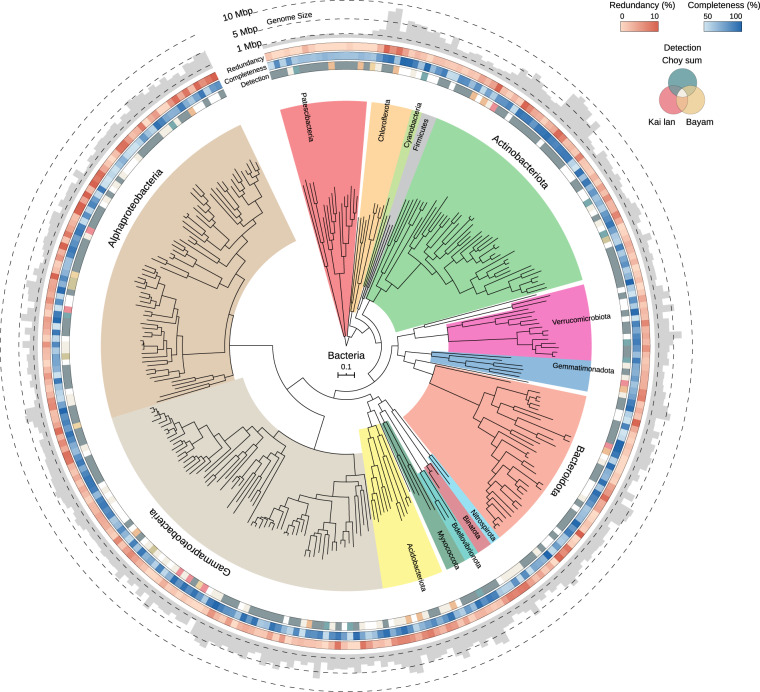
Maximum likelihood tree of all bacterial species-level MAGs. Phylogenetic tree constructed using a concatenated alignment of 120 conserved bacterial markers. Concentric rings moving outward from the tree show the detection of a MAG across the three vegetable crops computed globally, its completeness, and its redundancy respectively. The outermost bar plot shows the size of the MAG.

Here, we present 910 metagenome-assembled genomes (MAGs) reconstructed from 107 metagenomes, each of which represents a snapshot of the microbial communities sampled from different niches in the phytobiomes of three leafy Asian greens – *Brassica rapa* var. *parachinensis* (commonly known as Choy sum or Cai xin), *Brassica oleracea* var. *alboglabra* (commonly known as Kai lan) and *Amaranthus* spp. (commonly known as Bayam) – across their seedling and adult developmental stages. Sampled niches span the above-ground (phyllosphere) and below-ground (rhizosphere, rhizoplane, and endosphere) compartments of the crop-specific phytobiomes. Nearly two-thirds of the genomes are substantially complete with a completeness ≥70% and redundancy <10%, while the rest have a completeness ≥50% and redundancy <10%. However, several MAGs lack the full complement of rRNA genes owing to well-known challenges in assembling them from metagenomes^14,15^. The draft genomes cluster into 292 bacterial and 3 archaeal species-level groups, operationally defined based on 95% average nucleotide identity. A vast majority of them belong to the phyla Proteobacteria, Actinobacteria and Bacteroidetes (Fig. 1), which are known to be abundant across a wide-range of plant-associated microbiomes^10^. However, no eukaryotic genomes were recovered presumably due to limited sequencing depth. This collection also includes a subset from several underrepresented genus-level lineages in the Genome Taxonomy Database (GTDB) release 89 (r89) (Table 1).

**Table 1:** Phylogenetic diversity and gain for select bacterial genera. Top 15 bacterial genera with substantial phylogenetic gains relative to genomes from the GTDB r89. Phylogenetic diversity (PD) and phylogenetic gain (PG) were assessed using the bacterial domain-specific tree inferred using the concatenation of 120 conserved bacterial markers.

Genus	Number of Genomes		Relative Taxon PD (%)		PG (%)
	GTDB	This Study	GTDB	This Study	
UBA5195	2	2	53.66	53.43	46.34
Novosphingobium_A	4	3	55.76	54.29	44.24
12-FULL-67-14b	2	1	66.11	42.26	33.89
Streptomyces_C	2	1	67.25	40.46	32.75
UBA2020	3	1	67.78	39.39	32.22
Opitutus	5	4	71.61	50.11	28.39
SG8-41	2	1	72.44	42.41	27.56
UBA1487	3	1	73.35	33.84	26.65
Dokdonella	9	4	74.39	36.21	25.61
Bordetella_B	4	2	76.28	52.53	23.72
Pseudolabrys	6	3	76.79	41.9	23.21
C7867-002	5	1	79.94	22.25	20.06
Micavibrio_A	3	1	81.9	30.48	18.1
Lacunisphaera	5	2	82.06	26.63	17.94
UBA11358	9	1	84.5	29.44	15.5

We expect our collection of MAGs, together with the metagenomes from which they were recovered, to be useful for addressing a wide range of both fundamental and applied research questions concerning the various functional aspects of leafy vegetable phytobiomes. They are also likely to be useful for a range of comparative studies seeking to understand, among others, the genomic basis of microbe-plant relationships and the evolutionary context of individual genes, especially those related to the provisioning of plant-beneficial services. Finally, they may also offer clues for improving cultivation strategies for certain microbial species that lack cultured representatives.

## Methods

### Farm and management practices

Plant, soil and compost samples were collected from an intensively managed, soil-based commercial farm located in Lim Chu Kang, Singapore. A variety of horticultural crops, including leafy Asian greens, have been cultivated in this farm for nearly three decades. The farm produces on an average 300 tons per year of leafy Asian greens. Bayam and Kai lan were grown in the same greenhouse whereas Choy sum was grown in a different greenhouse. Bayam and Choy sum seeds were directly sown in soil (39 plants m^−2^) whereas Kai lan seeds were germinated in a nursery within the same farm. The nursery comprised of vertically stacked polypropylene seedling trays in which one seed was sown in each compost-filled cavity. Kai lan seedlings were on an average 15–20 days old when they were transplanted into the greenhouse soil bed. All three crops were on an average 30–45 days old with individual plants weighing 70–80 mg at the time of harvest.

Plants grown in the greenhouses were exposed only to sunlight and were irrigated using overhead water sprinklers on a daily basis. They were supplied with macronutrients through a one-time application of NPK (Nitrogen, Phosphorous and Potassium) fertilizers (1 kg m^−2^), approximately 1.5–2 weeks before harvest. Micronutrients were only applied when plants showed signs of deficiency. Microbial products were not used at any stage. Post-harvest, plants that did not meet the quality requirements for consumption were used for producing compost by allowing them to decompose in large pits. This compost was applied to the greenhouse bed before planting the next batch of crops.

### Sampling and sample processing

Adult plants which were ready for harvest were collected on 14 March 2018. A total of four lines from each soil bed were randomly chosen, from which one plant was randomly selected for sampling. Overall, 12 adult plant samples (four replicate plants per crop type) were collected in this manner. Plants were manually extricated in a gentle manner so that roots with any attached soil remained intact as much as possible. They were then stored in sterile, air-tight plastic bags and placed in an ice box. A total of 12 seedlings were sampled in a similar manner on 2 October 2018 and were on an average 15–20 days old at the time of collection. Kai lan seedlings were collected from four randomly chosen seedling trays. On both occasions, approximately 250 g of bulk soil from each greenhouse bed and compost samples from the seedling trays were collected using sterile plastic shovels and stored in a manner similar to the plant samples. Microbial cells from the phyllosphere and the rhizocompartments (rhizosphere, rhizoplane, and the endosphere) were isolated from each plant sample within 12 h from sample collection, using previously described protocols^9,16^. Cell pellets as well as aliquots of bulk soil and compost samples were then flash frozen using liquid nitrogen and stored at −80 ^ο^C until DNA extraction.

### Metagenomic DNA sequencing and assembly

Genomic DNA was extracted from all the samples using the ZymoBIOMICS DNA miniprep kit (Zymo Research, Irvine, CA, USA). Sequencing libraries were prepared and sequenced at the Singapore Center for Environmental Life Sciences Engineering genomics facility. Paired-end libraries (2 × 250 bp) were prepared using the TruSeq DNA library preparation kit (Illumina, San Diego, CA, USA) and sequenced on the HiSeq 2500 platform (Illumina, San Diego, CA, USA).

Raw demultiplexed reads were processed using Cutadapt v2.3^17^ with parameters: --error-rate 0.2, --minimum-length 75, --no-indels to remove sequencing adapters and BBDuk v38.56 (sourceforge.net/projects/bbmap/) with parameters: trimq=20, qtrim=rl, minlen=75 to trim low-quality regions.

Samples were de novo assembled both individually and by co-assembling those from the same niche or plant organ in a plant-type and growth stage specific manner using MEGAHIT v1.2.8^18^ with parameters: --k-min 27, --k-max 197, --k-step 10. Assembled contigs <1 kbp were discarded. Read containment was estimated by mapping the quality trimmed reads used for each assembly to the assembled contigs using Bowtie2 v2.3.5^19^ with parameters: --no-unal, -X 1000 and SAMtools^20^. Summaries of individual samples, assemblies including sample groupings for the co-assemblies, contigs >1 kbp from the individual and co-assemblies are available on figshare^21^ and are contained in the files “glv_sample_data.tsv”, “glv_asm_summary.tsv”, “glv_single_sample_asm.tar.gz” and “glv_co_asm.tar.gz” respectively.

### Genome binning, decontamination and dereplication

Contigs were clustered into metagenomic bins using MetaBAT2 v2.12.1^22^ with parameters: --minS 80, CONCOCT v1.1.0^23^ with parameters: -l 2500 and MaxBin2 v2.2.7^24^ with parameters: -min_contig_length 2500, all of which use a combination of sequence composition and differential coverage information. The latter was generated by mapping quality trimmed reads from individual samples to the contigs from each assembly using Bowtie2 v2.3.5^19^ with parameters: --no-unal, -X 1000 and SAMtools^20^, the results of which were processed using the jgi_summarize_bam_contig_depths script from MetaBAT2 v2.12.1^22^. Samples used for mapping comprised those that were used to generate a particular assembly as well as those expected to have similar microbial populations albeit at varying abundances. Multiple bins recovered from the same microbial population contained within a particular assembly were then aggregated and dereplicated using DAS Tool v1.1.0^25^ with parameters: --score_threshold 0.

Bins were refined by removing contigs with divergent genomic properties using RefineM v0.0.25^15^, and then using marker and reference-based approaches implemented in MAGPurify v1.0^26^. Contigs in each bin were removed if either their GC content or tetranucleotide distance fell outside the 98^th^ percentile of their expected distributions derived empirically from a highly curated set of genomes. Contigs were also removed if the absolute percentage difference between their mean coverage and the mean coverage of the bin was ≥ 50%. MAGpurify v1.0^26^ was then used to identify and remove taxonomically discordant contigs using the phylo-markers and clade-markers modules as well as contigs that aligned poorly to conspecific genomes from the IGGdb database^26^, when available, using the conspecific module. Finally, contigs that mapped to the nearest plant genomes from the Phytozome database v12.1^27^ (*Brassica rapa FPsc*, *Brassica oleracea capitata* and *Amaranthus hypochondriacus*), to those plant species included in this study, were removed using the known-contam module. A summary of the quality of the 910 MAGs before and after decontamination is available on figshare^21^ as “glv_mags_decontam_summary.tsv”. Decontaminated MAGs were then dereplicated using dRep v2.2.3^28^ with parameters: -comp 50, -con 10, -sani 0.95/0.99, --S_algorithm gANI.

### Genome quality assessment

**Fig. 2:**
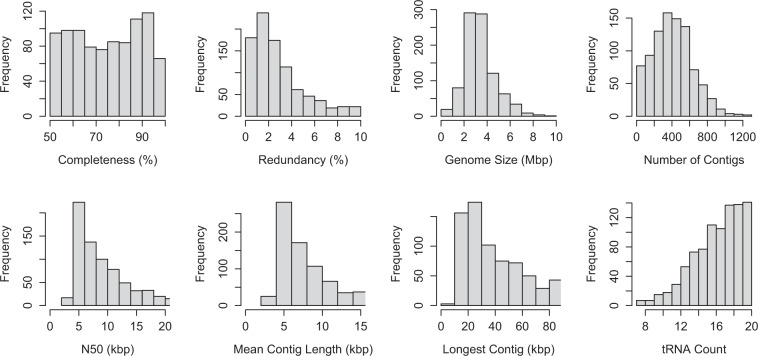
Quality metrics for the 910 MAGs. Data for contig N50, mean contig length and longest contig include only those that lie within the interquartile range. For the complete dataset, please refer to the table "glv_mags_qual_tax_summary.tsv" available on figshare^21^.

Assembly statistics for the 910 MAGs such as completeness, redundancy, size, number of contigs, contig N50, length of the longest contig, average GC content and the number of predicted genes were computed using the lineage workflow from CheckM v1.0.18^29^ and are summarized in Fig. 2. Transfer RNA gene sequences were predicted using tRNAScan-SE v2.0.5^30^ using the domain-specific models. MAGs were designated as near-complete drafts if they had a completeness >90%, redundancy <5% and transfer RNA gene sequences for at least 18 unique amino acids or as medium-quality drafts if they had a completeness ≥50% and a redundancy <10%. A summary of the assembly statistics for the 910 MAGs is available on figshare^21^ as “glv_mags_qual_tax_summary.tsv”.

### Detection of MAGs across samples

MAGs were detected across samples by mapping quality trimmed reads from all the samples to each MAG using Bowtie2 v2.3.5^19^ with parameters:--no-unal, -X 1000 and SAMtools^20^. The sample-specific mean coverage of each MAG was then computed using CoverM v0.4.0 (https://github.com/wwood/CoverM) with parameters: --min-read-percent-identity 0.95, --min-read-aligned-percent 0.75, --proper-pairs-only, --methods trimmed_mean. Coverage profiles were converted to global presence or absence across different vegetable crop types using R v.4.0.0^31^.

### Taxonomic classification and calculation of phylogenetic gain

The taxonomy of the 910 MAGs were inferred using GTDB-Tk v1.0.2^32–38^ with the GTDB r89^39,40^. This was cross-referenced with that inferred using 16S rRNA gene sequences, which were identified and extracted using the 16SfromHMM.py script (https://github.com/christophertbrown/bioscripts) from the ctbBio python package with parameters: -l 250 -m. Insertions ≥10 bp were removed using the strip_masked.py script from the same package with parameters: -l 10. Sequences were classified up to the genus level using the assignTaxonomy function with parameters: tryRC=TRUE, outputBootstraps=TRUE, while species labels were inferred using the addSpecies function from the DADA2 R package v1.14.1^41^. The reference database used for classification comprised of 20,486 bacterial and 1,073 archaeal full-length 16S rRNA gene sequences extracted from the set of representative species-level genomes in the GTDB r89^42^. Taxonomy inferred using both approaches and the full set of 16S rRNA gene sequences extracted from the MAGs are available on figshare^21^ and are contained in the files “glv_mags_qual_tax_summary.tsv”, “glv_mags_16SrDNA_tax.tsv” and “glv_mags_16SrDNA_seq.fa” respectively.

Phylogenetic relationships among the 292 bacterial species-level MAGs were inferred by constructing a maximum-likelihood tree using the de novo workflow in GTDB-Tk v1.2.0^32–38^ with parameters: --bacteria, --skip_gtdb_refs and --outgroup_taxon p_Patescibacteria. The tree was rooted on the branch leading to the MAGs from the phylum Patescibacteria. The rooted tree was visualized and annotated with data corresponding to MAG completeness, redundancy, size and detection across plant types using iTOL v5.5.1^43^. The unrooted version of this tree with bootstrap support values is available on figshare^21^ as “glv_mag_de_novo_unrooted.tree”. Domain-specific trees incorporating the 295 MAGs with species-level reference genomes from the GTDB r89^39,40^ were constructed using the de novo workflow in GTDB-Tk v1.2.0^32–38^ with parameters: --bacteria, --outgroup_taxon p_Patescibacteria and --archaea, --outgroup_taxon pAltiarchaeota for the bacterial and archaeal trees respectively. These trees were used to calculate the phylogenetic gain at different taxonomic levels using the pd_clade routine in genometreetk v0.1.6 (https://github.com/dparks1134/GenomeTreeTk). The unrooted, bootstrapped versions of the bacterial and archaeal trees are available on figshare^21^ and are contained in the files “glv_bac_de_novo_gtdb_unrooted.tree” and “glv_arc_de_novo_gtdb_unrooted.tree” respectively.

## Data Records

The raw sequence data is available on the NCBI Sequence Read Archive^44^. Datasets and data products generated from the raw sequence data are available in figshare^21^. They have been appropriately specified in the text where required.

## Technical Validation

This catalog comprises of only those genomes that met specific quality thresholds as described in the manuscript. Additionally, the taxonomy of MAGs inferred using whole-genome based methods were cross-referenced with those inferred using the 16S rRNA gene sequences, when available.

## Data Availability

Custom scripts were not used to generate or process this dataset. Software versions and non-default parameters used have been appropriately specified where required.
